# Fragile and conflict affected states: report from the Consultation on Collaboration for Applied Health Research and Delivery

**DOI:** 10.1186/1752-1505-8-15

**Published:** 2014-09-08

**Authors:** Joanna Raven, Tim Martineau, Eleanor MacPherson, Amuda Baba Dieu-Merci, Sarah Ssali, Steve Torr, Sally Theobald

**Affiliations:** 1 Department of International Public Health, Liverpool School of Tropical Medicine, Pembroke Place, Liverpool L3 5QA, UK; 2 Panafrican Institute of Community Health (IPASC), Bunia, Democratic Republic of Congo; 3 School of Women and Gender Studies, Makerere University, Makerere, Uganda; 4 Department of Vector Biology, Liverpool School of Tropical Medicine, Pembroke Place, Liverpool L3 5QA, UK

**Keywords:** Fragile and conflict affected states, Health systems, Human resources, Collaboration

## Abstract

Fragile and Conflict Affected States present difficult contexts to achieve health system outcomes and are neglected in health systems research. This report presents key debates from the Consultation of the Collaboration for Applied Health Research and Delivery, Liverpool, June, 2014.

## Introduction

The Collaboration for Applied Health Research and Delivery (CAHRD) is a global network which actively promotes and sustains multi-disciplinary engagement in the co-production and utilization of new knowledge. The inaugural Consultation of CAHRD held in Liverpool, UK on the 12 and 13 June, 2014, brought together 200 delegates including researchers from different disciplines and policy makers from Africa, Asia and Europe. The purpose of the consultation was to foster dialogue across the CAHRD streams, through the development of multi-disciplinary papers and interactive discussions during the meeting in order to shape the strategic direction for CAHRD over the next 10 to 20 years. Papers were presented on the selected streams of lung health, maternal and newborn health and neglected tropical diseases (NTDs) and health systems including in the context of Fragile and Conflict Affected States (FCAS). These streams were debated in an external panel which critiqued the papers; small group discussions; and a TV-style Question Time debate open to the public.

In this report we highlight the key issues about health systems in the context of FCAS addressed during the consultation.

### Framing the debate: Human resources for health from the perspective of IPASC in rural Democratic Republic of Congo

The Institut Panafricain de Santé Communautaire et Medicine Tropicale (IPASC) is a faith based organisation in North Eastern Democratic Republic of Congo with the aim of improving population health through holistic and participatory approaches. Through their work with communities and health system, IPASC director, Mr. Amuda Baba Dieu-Merci, identified many human resource challenges. He brought home the risks that health workers face: a hospital south of Bunia was recently attacked by a rebel group as they thought they were treating government troops, killing 3 health care workers, and cutting the hospital off from essential supplies. “*People don’t work in rural areas like this as it can be too dangerous, they prefer to stay in bigger places where it is seen as safer - some people go there and leave their family in a safe place.”*

He further described how staff staying in the rural areas, face difficult working conditions such as: drug shortages as it is too dangerous to travel to the urban areas for supplies, poorly equipped health facilities, limited options for capacity building, not being able to transfer patients due to poor road infrastructure and lack of transport, limited supervision and working in a context of extreme poverty.

### Linking across the CAHRD consultation focal streams: What are the health systems challenges in addressing NTDs in FCAS?

The dialogue in this consultation illustrates the health systems and human resource issues in delivering health care services for NTDs in FCAS, an area where there is little published literature.

Both Lymphatic Filiariasis (LF) and Human African Trypanosomiasis (HAT) are particularly prevalent in FCAS. Health systems challenges with NTDs in FCAS included:

• Building robust human resource systems requires good leadership and investment which can be challenging in FCAS contexts where there are multiple unfolding priorities. Conflict or fragility in one setting can affect NTD programming in neighbouring non-conflict regions or countries as seen in HAT transmission in South Sudan and Northern Uganda. NTD directors not only have to forge alliances and working relationships across the health sector and other related sectors e.g. Agriculture in their own countries but also with neighbouring countries. Little is known about what approaches for developing leadership work well in these situations.

• Screening, diagnosis and treatment services during conflicts have largely been provided by NGOs. Following the withdrawal of NGOs, health workers are often re-integrated into the health system and are required to provide other services. Expertise in NTD specific diagnosis and treatment may deteriorate, and is difficult to restore. There is a need to better understand how to support and build sustainable capacity to deliver quality services in the post-conflict period.

• Much of the care for morbidity management takes place at community level. Providing care to people with long term infections such as lymphedema following LF infection requires skilled support from carers and health workers. Within households this caring role is normally the responsibility of women and girls although little is known about the realities and impact of this within FCAS. But with conflict, extreme poverty and health care shortages this is likely to be particularly challenging. We need to further understand how to strengthen the responsiveness of health systems in FCAS to support men and women living with lifelong disability and their carers.

### Building strong and responsive health systems in FCAS requires inter-sectoral action

Emerging as a key area for future action at the CAHRD meeting was the importance of inter-sectoral action. Fostering such an approach requires time and resources, and new ways of working, which is all the more challenging in FCAS with health workforce constraints and fluidity. Nevertheless, they may also present strategic opportunities to forge new partnerships and collaborations. One example highlighted in the debate was the issue of sexual and gender based violence (SGBV). In FCAS, responses to SGBV require both a strong health systems approach and appropriate linkages across other sectors e.g. education, gender and social welfare, in order to support survivors, build resilience and prevent future violence. In the Question Time debate, panellist Mr. Amuda Baba argued that inter-sectoral action is critical to taking forward health and addressing social determinants and that responses to SGBV require action across sectors. He gave an example from Bunia, DRC, where an interdisciplinary committee made up of representatives from local government, education, health, army, police and women’s groups was set up after the 2003 conflict, to address the issues of SGBV and was instrumental in developing a national law against sexual violence. This argument was reinforced by Dr. Sarah Ssali, ReBUILD researcher from the School of Women and Gender Studies at Makerere University, who also highlighted the urgent need to build new partnerships to reduce stigmatisation of women, girls, men and boy survivors of SGBV and stop ongoing violence.

### Fragility and conflict: partnership and action for the way forward

FCAS present particularly difficult contexts to achieve health system outcomes, such as better health, equity, social inclusion and trust. Fragility is not a constant status: countries, or parts of countries, may move in and out of fragile situations. This concept of fluidity, developing context specific responses and learning across contexts resonated with many delegates. Chris Whitty, Chief Scientific Advisor, DFID, further argued that FCAS are not going away soon, they have been neglected to date in health systems strengthening and research and are important contexts in which to better understand and build strong and equitable health systems.

Emerging from both the consultation and the Question Time debate was a strong call for:

• Local solutions to local issues, including supporting and building networks;

• Strengthening networks to support exchange, including south to south dialogue and action;

• New platforms, collaborations and ways of doing things; and

• Developing viable processes and approaches for research capacity strengthening in FCAS.

We plan to continue this dialogue through the Health Systems Global Thematic Working Group on Health Systems in Fragile and Conflicted Affected States which brings together key actors with interests in research, policy and advocacy to strengthen health systems in these states. Do join us!

## Competing interests

The authors declare that they have no competing interests.

## Authors’ contributions

JR drafted the manuscript. All authors contributed to revising the manuscript. All authors read and approved the final manuscript.

## Acknowledgments

This manuscript is part of the ‘Filling the Void: Health systems in fragile and conflict affected states’ thematic series.

